# A Novel Equation for Cooperativity of the Allosteric State Function^☆^

**DOI:** 10.1016/j.jmb.2013.09.010

**Published:** 2014-01-09

**Authors:** Stuart J. Edelstein

**Affiliations:** Babraham Institute, Cambridge CB22 3AT, UK

**Keywords:** allosteric, MWC model, Hill coefficient, state function, cooperativity

## Abstract

The MWC (*M*onod–*W*yman–*C*hangeux) allosteric model postulates concerted conformational changes between two states: the intrinsically more stable T state with relatively weak ligand binding and the R state with relatively strong ligand binding. The model distinguishes between $\bar{Y}$ (the fractional occupation of the binding sites) and $\bar{R}$ (the fraction of molecules in the R state). Cooperativity (measured by the Hill coefficient) has strikingly different properties for $\bar{Y}$ and $\bar{R}$. For the latter, cooperativity depends only on the relative affinities of the two states, not on their relative intrinsic stabilities, as demonstrated here with a simple new equation relating the Hill coefficient to $\bar{R}$.

## Highlights

• A simple new equation relating the Hill coefficient to $\bar{R}$ is presented.
• This equation shows that cooperativity (measured by the Hill coefficient) for $\bar{R}$ depends only on the relative affinities of the two states, not on their relative intrinsic stabilities.
• The curves for $\bar{R}$ may be characterized by Hill coefficients < 1, even under conditions of positive cooperativity.

## Graphical Abstract

**Figure:**
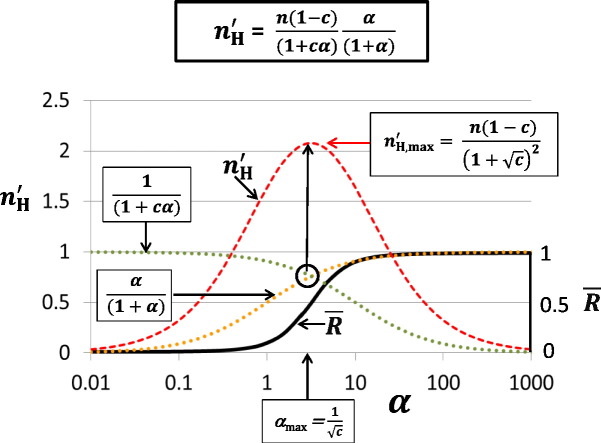

The concept of allosteric interactions, introduced a half-century ago [1], [2], [3], has had a powerful impact in biology for problems of signal transduction and control at various levels [4], [5], [6], [7]. The generalization of allostery is reflected by the fact that, some 50 years after creation of this neologism, “allosteric” as a keyword generates over 18,000 responses in PubMed. Surprisingly, however, a number of fundamental conceptual issues concerning allosteric cooperativity still require clarification. The original mathematical formulation in the MWC (*M*onod–*W*yman–*C*hangeux) model for allosteric proteins is based on two distinct conformational states (T and R) related by a single intrinsic equilibrium constant, *L*, where *L* = [T]/[R] in the absence of ligands for that protein [3]. Each state is characterized by its equilibrium dissociation constant for a particular ligand (*K*_R_ for the R state and *K*_T_ for the T state) at the *n* binding sites for an oligomer with *n* identical subunits. These simple definitions permit distinguishing between the binding function $\bar{Y}$ (the fraction of sites occupied for both states) and the state function $\bar{R}$ (the fraction of molecules in the R state):

(1) $$\bar{Y}=\frac{\alpha {\left(1+\alpha \right)}^{n-1}+\mathrm{Lc\alpha}{\left(1+\mathrm{c\alpha}\right)}^{n-1}}{{\left(1+\alpha \right)}^{n}+L{\left(1+\mathrm{c\alpha}\right)}^{n}}$$

(2) $$\bar{R}=\frac{{\left(1+\alpha \right)}^{n}}{{\left(1+\alpha \right)}^{n}+L{\left(1+\mathrm{c\alpha}\right)}^{n}}$$

Both equations are expressed with respect to *α*, the concentration of the ligand normalized to the affinity of the R state (*α* = [ligand]/*K*_R_) and the ratio of affinities for the R and T states given by *c* = *K*_R_/*K*_T_.

A general feature of oligomeric allosteric proteins is a sigmoidal curve for $\bar{Y}$ as a function of *α*, as established in the early investigations on the binding of oxygen to hemoglobin. The degree of cooperativity is conveniently described by *n*_H_, the Hill coefficient [8]. Although the equation for $\bar{Y}$ has been frequently employed in the context of the MWC model, a simple analytical expression for *n*_H_ at all values of $\bar{Y}$ from 0 to 1 had been lacking. Several different versions were published over the years, but they were exceeding complex [9], [10], [11]. An unexpectedly simple equation for *n*_H_ was presented in a manuscript by Crick and Wyman that circulated among a very limited number of scientists in 1965 but was never submitted for publication until rediscovered in my files and recently published [12]. In their original text, Crick and Wyman noted that, for their novel equation, “One naturally suspects that there is a simple derivation of it, but we were unable to discover it”. My colleagues and I were able to achieve a compact derivation, which was published at the same time [13]. This success encouraged me to seek a concise equation for the Hill coefficient of $\bar{R}$, which is presented here.

By analogy with $\bar{Y}$, the cooperativity of $\bar{R}$ for an oligomer with *n* subunits can be defined by a corresponding Hill coefficient ${n^{\prime }}_{H}$:

(3) $${n^{\prime }}_{H}=\frac{d\;\log\left(\frac{\bar{R}}{1-\bar{R}\;}\right)}{d\;\log\;\alpha }$$

Previously published solutions for the cooperativity of $\bar{R}$ involved complex sums with a number of terms equivalent to the number of subunits [14] or were applicable only to curves of $\bar{R}$ that were normalized [14], [15]. However, following an approach along the lines we employed for the derivation of the Crick–Wyman equation for the cooperativity of $\bar{Y}$ [13], I was able to obtain the following simple equation for the cooperativity for $\bar{R}$ based on the parameters of the MWC model:

(4) $${n^{\prime }}_{H}=\frac{n\left(1-c\right)}{\left(1+\mathrm{c\alpha}\right)}\frac{\alpha }{\left(1+\alpha \right)}$$

A full derivation is presented in Supplementary Material. By graphing the two principle fractions, as shown in Fig. 1a, this equation is readily understood in terms of increasing values of $\frac{\alpha }{\left(1+\alpha \right)}$ and decreasing values of $\;\frac{1}{\left(1+\mathrm{c\alpha}\right)}$ as a function of *α*. The intersection point of the two curves corresponds to the maximum of ${n^{\prime }}_{H}$ and occurs at a value of $\alpha =\frac{1}{\sqrt{c}}$. Since $\frac{\alpha }{\left(1+\alpha \right)}$ is equal to $\frac{1}{\left(1+\mathrm{c\alpha}\right)}$ at that point, Eq. (4) for ${n^{\prime }}_{H}$ at $\alpha =\frac{1}{\sqrt{c}}$ simplifies to:

(5) $${n^{\prime }}_{H,\max}=\frac{n\left(1-c\right)}{{\left(1+\sqrt{c}\right)}^{2}}$$

**Fig. 1:**
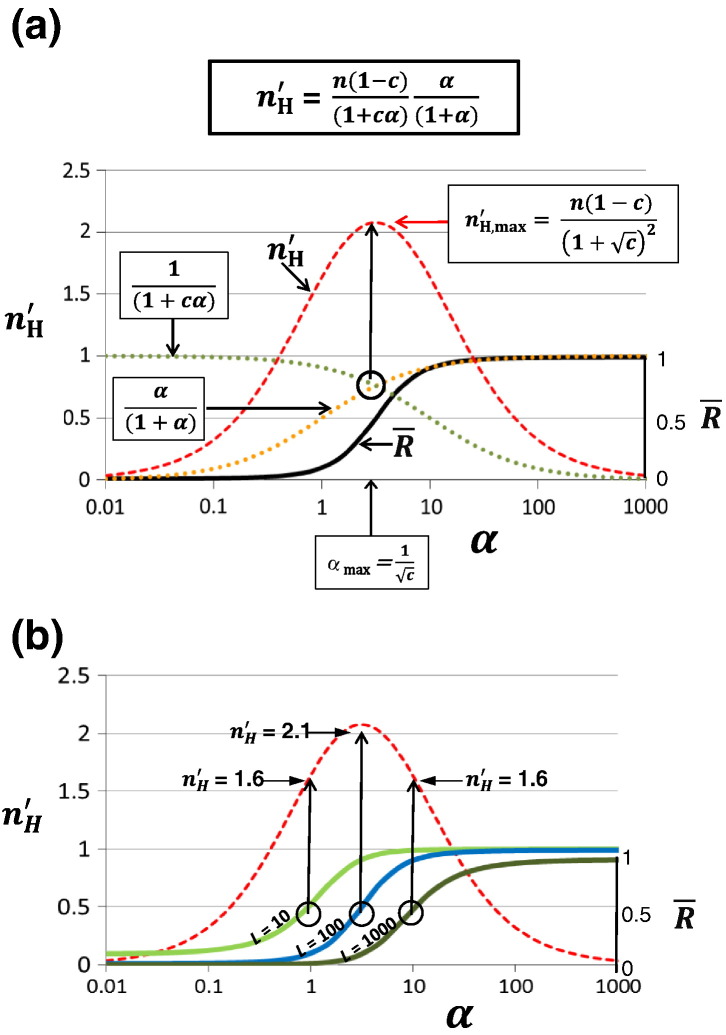
Graphical description of the equation describing the cooperativity of $\bar{R}$ in the context of the MWC model. (a) The basic equation is represented for ${n^{\prime }}_{H}$ (red broken line) on the left ordinate and $\bar{R}$ (black continuous line) on the right ordinate as a function of *α*. The curves are calculated for a tetramer (*n* = 4) with values of *L* = 100 and *c* = 0.1. The contributions to the properties of the basic Eq. (4) are graphed for $\frac{\alpha }{\left(1+\alpha \right)}$ and $\frac{1}{\left(1+\mathrm{c\alpha}\right)}$ as a function of *α* to show that the point of intersection of these two curves corresponds to ${n^{\prime }}_{H,\max}$. At that point, $\frac{1}{\left(1+\alpha \right)}=\frac{1}{\left(1+\mathrm{c\alpha}\right)}$, which converts to *α* + *cα*^2^ = 1 + *α*, and can be simplified to *cα*^2^ = 1. Hence, at that point, $\alpha_{\max}=\frac{1}{\sqrt{c}}$. Moreover, at *α*_max_, the basic Eq. (4) for ${n^{\prime }}_{H}$ reduces to ${n^{\prime }}_{H,\max}=n\left(1-c\right)\;{\left(\frac{\alpha_{\max}}{\left(1+\alpha_{\max}\right)}\right)}^{2}$. Since $\frac{\alpha_{\max}}{\left(1+\alpha_{\max}\right)}=\frac{\frac{1}{\sqrt{c}}\;}{1+\frac{1}{\sqrt{c}}}=\frac{1}{1+\sqrt{c}}$, it follows that ${n^{\prime }}_{H,\max}=\frac{n\left(1-c\right)}{{\left(1+\sqrt{c}\right)}^{2}}\;$. (b) Three curves for $\bar{R}$ at different values of *L* to illustrate how each value for ${n^{\prime }}_{H}$ at $\bar{R}=0.5$ are determined by the intersection of the value of *α* at that point (defined as *α*_50_) with the curve for ${n^{\prime }}_{H}$*versus α* fixed by Eq. (4).

The simplicity of the relations presented in Fig. 1a emphasizes an important feature of cooperativity for the state function $\left(\bar{R}\right)$, namely, that ${n^{\prime }}_{H}$ is independent of the allosteric constant, *L*, as previously noted [14], [15], [16]. As a consequence, the apparent value of ${n^{\prime }}_{H}$, for example, at the value of $\bar{R}=0.5$, is fixed only by the value of *α* at that point (designated *α*_50_) for a given value of *c*. This property is illustrated in Fig. 1b for three curves that differ only in their values of *L*. Since the dependence of ${n^{\prime }}_{H}$ on *α* is the same for the three curves (with *c* held constant), for each curve, the value of ${n^{\prime }}_{H}$ at $\bar{R}=0.5$ corresponds to the intersection of the value of *α*_50_ with the curve for ${n^{\prime }}_{H}$ determined by Eq. (4).

The article by Crick and Wyman served an additional useful purpose by developing the concept of the allosteric range [12]. A condition of the MWC model is that the transition between the T state and the R state is never totally complete. A fraction of molecules will always be present in the R state, even in the absence of ligand, and a fraction of molecules will be present in the T state, even at full saturation. The degree to which these fractions are significant depends on the values of *L* and *c*. The values of $\bar{R}$ between the minimum and the maximum encompass the allosteric range, abbreviated as *Q* [12], where ${\bar{R}}_{\min}=\frac{1}{1+L}$ and ${\bar{R}}_{\max}\frac{1}{1+Lc^{n}}$, with the allosteric range defined by $Q={\bar{R}}_{\max}-{\bar{R}}_{\min}$. Early applications of this concept were made by Rubin and Changeux [17]. In general, low values of *L* and high values of *c* (approaching *c* = 1) will tend to give values of *Q* ≪ 1.

The $\bar{R}$ curves can be normalized to a range 0–1 to give ${\bar{R}}_{\mathrm{norm}}$, calculated as ${\bar{R}}_{\mathrm{norm}}=\frac{\bar{R}-{\bar{R}}_{\min}}{Q}$. It is apparent that the sigmoidal character of the curves for $\bar{R}$ may be significantly enhanced by normalization. As a result of this stretching in the vertical dimension for ${\bar{R}}_{\mathrm{norm}}$ compared to $\bar{R}$, sigmoidality is exaggerated and the apparent Hill coefficient increases. Hence, quantitative estimates of cooperativity are clearly influenced by normalization. Qin obtained an equation for the Hill coefficient of the normalized state function $\left({n^{\prime }}_{H,\mathrm{norm}}\right)$ [15] but used unconventional nomenclature, which impedes comparison to the usual formulations of the MWC model. However, after converting it into classical MWC terminology and rearranging, his equation (number 6 of his article [15]) can be expressed as:

(6) $${n^{\prime }}_{H,\mathrm{norm}}=\frac{n\left(1-c\right)}{\left(1-\mathrm{c\alpha}\right)}\frac{\alpha }{\left(1-\alpha \right)}\frac{1-c^{n}}{\left\{{\left[\frac{c\left(1+\alpha \right)}{1+\mathrm{c\alpha}}\right]}^{n}-1\right\}\left\{{\left[\frac{\left(1+\mathrm{c\alpha}\right)}{1+\alpha }\right]}^{n}-1\right\}}$$

Since Eq. (6) is composed of three fractions of which the first two correspond to Eq. (4) for the Hill coefficient of $\bar{R}$ without normalization, the third fraction can be used as a conversion factor to convert the value of ${n^{\prime }}_{H}$ to ${n^{\prime }}_{H,\mathrm{norm}}$. Hence, the Hill coefficient $\left({n^{\prime }}_{H,\mathrm{norm}}\right)$ for any ${\bar{R}}_{\mathrm{norm}}$ can be obtained by multiplying the value of ${n^{\prime }}_{H}$ for non-normalized $\bar{R}$ from Eq. (4) by the normalization conversion factor:

(7) $$\frac{1-c^{n}}{\left\{{\left[\frac{c\left(1+\alpha \right)}{1+\mathrm{c\alpha}}\right]}^{n}-1\right\}\left\{{\left[\frac{\left(1+\mathrm{c\alpha}\right)}{1+\alpha }\right]}^{n}-1\right\}}$$

Overall, the smaller the value of *Q*, the greater the consequences of normalization with respect to the apparent cooperativity. Since cooperativity is independent of *L*, the effect of normalization is sensitive only to *c*. For values of *c* < 0.1, the effect of normalization on cooperativity is negligible, but for values of *c* in the range 0.1–1.0, the effects of normalization are significant and increase dramatically as *c* approaches 1.0.

The results presented here emphasize that the cooperativity of $\bar{R}$ is fundamentally different from the cooperativity of $\bar{Y}$. Values of *n*_H_ < 1 are considered to be a sign of negative cooperativity for $\bar{Y}$, but the same reasoning does not apply to $\bar{R}$. Under many conditions, cooperative oligomers exhibit values of ${n^{\prime }}_{H}<1$ for $\bar{R}$, particularly at relatively high values of *c* [16]. In this case, *Q* < 1 and applying normalization can increase values to ${n^{\prime }}_{H}>1$.

Examples showing the importance of estimating the Hill coefficient correctly for $\bar{R}$, as well as the significance of normalization, can be deduced both from the classical literature on allosteric enzymes and in the more recent literature for allosteric receptors. Concerning enzymes, critical experiments on aspartate transcarbamylase demonstrated distinct dependences of $\bar{Y}$ and $\bar{R}$ on the concentration of a substrate analog [18], with a value of the Hill coefficient reported for $\bar{Y}\;\left(n_{H}=1.55\right)$, but none reported for $\bar{R}$. However, by applying the principles of cooperativity to $\bar{R}$ along the lines described here, a Hill coefficient for $\bar{R}$ of ${n^{\prime }}_{H}=1.12$ could be obtained from the original data [16]. Hence, for concurrent measurements under the same conditions, $\bar{Y}$ and $\bar{R}$ may display significantly different levels of cooperativity. Concerning allosteric membrane receptors, the widely studied neuronal nicotinic receptor α7, a cooperative homopentamer [19], displays curves for $\bar{R}$ under standard conditions characterized by a Hill coefficient of ${n^{\prime }}_{H}=0.14$, a surprisingly low value [20], but normalization results in a large increase in the estimated value. Effects of normalization are particularly striking for G-protein-coupled receptors, since the relevant allosteric range is frequently characterized by a value of *Q* ≪ 1, but the data are generally presented after normalization [20]. In contrast to $\bar{Y}$, which is unaffected by normalization, the properties of $\bar{R}$ are particularly sensitive to normalization.

It came to my attention after this Brevia was in press that D. Colquhoun had published an equation for the maximum value of the Hill coefficient for normalized curves of $\bar{R}$ in a book chapter [21]. His equation (originally published as a ratio of sums, but presented here in the compact equivalent form he sent to me) is:

$${n^{\prime }}_{H,\max}=\frac{n\left(1-c\right)\left(1-c^{n}\right)}{{\left(1+\sqrt{c}\right)}^{2}{\left(1-\sqrt{c^{n}}\right)}^{2}}$$

Interestingly, this equation covers a case that was not treated in the Brevia, but it yields a value that can be obtained by multiplying Eqs. (5) and (7) of the Brevia, when $\alpha =\frac{1}{\sqrt{c}}$, the concentration at which the ${n^{\prime }}_{H,\max}$ occurs.

## Appendix A Supplementary data

Derivation of the equation for the Hill coefficient of the state function.
