# Supplementary material for: A Novel Equation for Cooperativity of the Allosteric State Function
Source: J Mol Biol. 2014 Jan 9;426(1):39–42. doi: 10.1016/j.jmb.2013.09.010 (PMC3898861; doi:10.1016/j.jmb.2013.09.010)
Supplement: Supplementary file 1 — Derivation of the equation for the Hill coefficient of the state function. [file mmc1.pdf]

Supplementary Material

BREVIA

A novel equation for cooperativity of the allosteric state function

Stuart Edelstein

Babraham Institute, Cambridge CB22 3AT, UK.

Derivation of the equation for the Hill coefficient of the state function. By analogy with $\bar{Y}$, the Hill coefficient for $\bar{R}$, here represented by n'_H, can be defined as:

$$n'_{\mathrm{H}} = \frac{d\,log\left(\frac{\bar{R}}{1-\bar{R}}\right)}{d\,log\,\alpha} = \frac{\alpha}{\bar{R}\,(1-\bar{R})}\left(\frac{d\,\bar{R}}{d\,\alpha}\right) \tag{S1}$$

where $\bar{R}$ is defined by the Monod-Wyman-Changeux equation for an oligomer with n subunits:[1]

$$\bar{R} = \frac{(1+\alpha)^n}{(1+\alpha)^n + L(1+c\alpha)^n} \tag{S2}$$

In order to replace the terms in the denominator on the right side of equation (S1), the term $1-\bar{R}$ is recast as:

$$1-\bar{R} = \frac{(1+\alpha)^n + L(1+c\alpha)^n - (1+\alpha)^n}{(1+\alpha)^n + L(1+c\alpha)^n} = \frac{L(1+c\alpha)^n}{(1+\alpha)^n + L(1+c\alpha)^n} \tag{S3}$$

Combining terms in equations (S2) and (S3) for $\bar{R}\,(1-\bar{R})$ gives:

$$\bar{R}\,(1-\bar{R}) = \frac{(1+\alpha)^n\,L(1+c\alpha)^n}{[(1+\alpha)^n + L(1+c\alpha)^n]^2} \tag{S4}$$

For the term $\frac{d\,\bar{R}}{d\,\alpha}$, use is made of the equation previously reported:[2]

$$\frac{d\,\bar{R}}{d\,\alpha} = \frac{nL\left(\frac{1+c\alpha}{1+\alpha}\right)^{n-1}(1-c)}{\left[1+L\left(\frac{1+c\alpha}{1+\alpha}\right)^n\right]^2(1+\alpha)^2} \tag{S5}$$

Incorporating the terms in equations (S4) and (S5) into equation (S1) yields the following equation for n'_{H}:

$$n'_H = \frac{\alpha[(1+\alpha)^n + L(1+c\alpha)^n]^2}{(1+\alpha)^n\,L(1+c\alpha)^n}\,\frac{nL\left(\frac{1+c\alpha}{1+\alpha}\right)^{n-1}(1-c)}{\left[1+L\left(\frac{1+c\alpha}{1+\alpha}\right)^n\right]^2(1+\alpha)^2} \tag{S6}$$

Equation (S6) can be rearranged to a form that facilitates cancellation of terms:

$$n'_{\mathrm{H}} = \frac{n(1-c)L(1+c\alpha)^{n-1}}{L(1+c\alpha)^{n}} \frac{\alpha[(1+\alpha)^{n} + L(1+c\alpha)^{n}]^{2}}{[(1+\alpha)^{n}+L(1+c\alpha)^{n}]^{2}} \frac{(1+\alpha)^{n}\,(1+\alpha)^{n}}{(1+\alpha)^{n-1}(1+\alpha)^{2}(1+\alpha)^{n}} \tag{S7}$$

Upon cancellation of identical terms in the numerator and the denominator, the simple equation (4) in the main text for the Hill coefficient of $\bar{R}$ is obtained:

$$n'_{\mathrm{H}} = \frac{n(1-c)}{(1+c\alpha)} \frac{\alpha}{(1+\alpha)} \tag{S8}$$

Reference:

1. Monod, J., Wyman, J. & Changeux, J.-P. (1965). On the nature of allosteric transitions: A plausible model. *J. Mol. Biol.* **12**, 88-118.
2. Edelstein, S. J., Stefan, M. I. & Le Novere, N. (2010). Ligand depletion in vivo modulates the dynamic range and cooperativity of signal transduction. *PLoS One* **5**, e8449.
